# Adolescent girls’ academic support-seeking, depression, and anxiety: the mediating role of digital support-seeking

**DOI:** 10.1080/00049530.2023.2170279

**Published:** 2023-02-08

**Authors:** Erin Mackenzie, Anne McMaugh, Penny Van Bergen, Roberto H. Parada

**Affiliations:** a Macquarie School of Education, Macquarie University; b Centre for Educational Research, Western Sydney University; c School of Education, University of Wollongong

**Keywords:** Digital support seeking, coping, depression, anxiety, academic stress

## Abstract

**Objective:**

This study explored how seeking support from friends and parents and informal digital sources are related to anxiety and depression in adolescent girls.

**Method:**

Early and middle adolescent girls (*N* = 186) were presented with four vignettes of academic stressors; for each scenario, they rated their likelihood of seeking support from parents, friends, or digital sources. Depression and anxiety symptoms were measured using the youth version of the Depression, Anxiety, and Stress Scale. Alternate models were tested using Structural Equation Modelling.

**Results:**

Digital support seeking mediated the relationships between seeking support from parents and friends and anxiety and depression. Seeking support from parents was negatively related to digital support seeking, which in turn was positively related to depression and anxiety. In contrast, seeking support from friends was positively related to digital support seeking.

**Conclusion:**

These findings suggest that informal digital support seeking may be considered a problematic way of coping with academic stress for adolescent girls, while seeking support from parents can be considered a protective factor due to its negative relationship with digital support-seeking.

## KEY POINTS

**What is already known about this topic:**

(1) Most adolescents use online communication on a daily basis to connect with friends, which provides unprecedented access to seeking informal academic support from these friends.
(2) While seeking support is generally viewed as an adaptive coping strategy for adolescents, there is emerging evidence to suggest that online coping is related to poorer mental health. However, it is unknown how digital support seeking for academic stressors is related to depression and anxiety.
(3) Adolescent girls are more likely than boys to seek support from others and are also more likely to experience depression and anxiety.

**What this topic adds:**

(1) The current study is the first to examine relationships between digital support seeking, seeking support from traditional sources (parents and friends), and depression and anxiety in adolescent girls.
(2) Digital support-seeking demonstrated relationships with higher indicators of depression and anxiety, indicating it is a problematic way of coping with academic stress. We propose seeking support from parents as a protective factor due to its negative relationship with digital support-seeking.
(3) Given the salience of academic stressors for adolescents, the findings suggest that parents, teachers, and practitioners should be wary of maladaptive outcomes when adolescents seek help online for academic concerns.

Academic stressors are a normative aspect of adolescents’ lives (Moksnes et al., 2016) and the ability to employ adaptive coping responses is crucial to avoid negative outcomes experienced in response to school stress (Evans et al., 2018; Plenty et al., 2014). Adolescents report academic concerns and schoolwork among their top stressors (Arbel et al., 2018). The ways in which adolescents respond to this stress are important, with adaptive responses implicated in healthy psychosocial development (Compas et al., 2001; Skinner et al., 2003). Support-seeking is a common strategy used by adolescents to cope with stressors (Skinner et al., 2003) and can reduce the adverse effects of academic stress (Sotardi et al., 2021).

Although support-seeking is typically considered adaptive (Arbel et al., 2018), findings related to adolescents’ mental health are equivocal. Chu et al. (2010) reported a weak positive association between adolescent support-seeking and wellbeing, while Heerde and Hemphill (2018) found no relationship with internalising symptoms. These equivocal findings may reflect the multifaceted nature of support-seeking. Adolescents may seek support from teachers but are more likely to seek support from parents and friends (Sotardi et al., 2021). In addition, while research has traditionally focused on face-to-face support-seeking, some adolescents cope with negative experiences by seeking support online (Frison & Eggermont, 2015). Below we consider adolescent mental health in light of the sources of support adolescents draw on when facing academic stressors and the way in which they do so.

## Sources of support

When considering the source of support, there is some evidence to suggest that seeking support from parents in early to mid-adolescence has a greater impact on wellbeing than seeking support from peers. For example, Heerde and Hemphill’s (2018) meta-analysis found that seeking help from parents or a combination of parents and peers was associated with improved psychosocial outcomes compared to other sources of support such as school personnel and other significant adults. Similarly, Szwedo et al. (2017) reported that seeking support from parents at age 13 years predicted functional independence at 25 years. Research also suggests that the perception of social support from parents is also a stronger predictor of wellbeing than perceived peer support across adolescence (Seiffge-Krenke & Persike, 2017; Wentzel et al., 2016). Despite these findings, seeking support from friends is also critical. Reflecting the increasing importance of peers in adolescence (Albert et al., 2013), support from friends appears to increasingly reduce physiological stress during adolescence, while the stress buffering effects of parental support decreases (Gunnar & Hostinar, 2015). Taken together, these findings suggest that seeking support from both parents and friends is likely to be an adaptive way of coping in adolescence.

## Traditional and digital support seeking

While studies of social support have traditionally focused on face-to-face support from parents, friends, and peers (Rickwood et al., 2005; Wilson et al., 2005), it is increasingly evident that some adolescents seek informal support online as well as in-person. For example, Duvenage et al. (2020) found that adolescents cope with negative experiences by seeking emotional support online, in addition to using technology as a distraction and to obtain information. Furthermore, adolescents who experience higher levels of daily stress have been found to be more likely to seek support through Facebook (Frison & Eggermont, 2015).

Digital support is typically sought from known contacts (Mackenzie et al., 2020), yet the quality and outcomes of support offered via digital means may vary from that offered face-to-face. For example, digital support may be less effective than in-person support due to reduced social cues, which may undermine the clarity, genuineness or emotional relief of the support provided (Rains et al., 2016; Vermeulen et al., 2018; Walther et al., 2015).

While the mental health implications of adolescents’ digital support-seeking for academic stressors are unknown, adolescents’ use of online coping for everyday stressors has been associated with worry, jealousy, and loneliness (Duvenage et al., 2020). Higher levels of emotional support-seeking on Facebook have also been related to depressed mood (Frison & Eggermont, 2015). The constancy of contact afforded by digital communication may also mean that adolescents have a heightened expectation that help is always available (Nesi et al., 2018). In the context of online academic help seeking, adolescent girls have identified that this availability allows them to ask for help with homework and for answers to homework whenever they need it (Mackenzie et al., 2020). While this was perceived as beneficial by adolescents, it is quite possible that a quick deferral to friends for help with homework before trying to solve a problem leads to poorer understanding and achievement (Ryan & Shim, 2012), each of which could contribute to poorer mental health.

## Current study

This study explored adolescent girls’ digital and traditional support-seeking intentions in response to everyday academic stressors. Adolescent girls are more likely than boys to seek support from others (Sotardi et al., 2021) and to experience anxiety and depression (Ohannessian et al., 2017; Salk et al., 2017). Given that support-seeking intentions predict real-world behaviours (Nagai, 2015), we used vignettes to capture relationships between support seeking intentions (digital, from parents, or from friends) and mental health symptoms (depression and anxiety). As adolescents predominantly use online communication with friends (Mackenzie et al., 2020), we expected those who reported greater intentions to seek support from friends would also do so digitally. However, we predicted different relationships with mental health. Based on the extant literature, we expected that intentions to seek parents’ and friends’ support for academic stressors would be negatively related to depression and anxiety (Sotardi et al., 2021) and that digital support-seeking would be positively related (Duvenage et al., 2020; Frison & Eggermont, 2015). We used structural equation modelling to explore relations between and among these sources and the mental health indicators including if one or other support source might play a mediating role in the effectiveness of the other sources.

## Method

### Participants

The participants were 186 girls from four independent girls’ schools in Sydney, Australia, including 78 girls in Grade 7 (*M*_age_ = 12.55 years, *SD* = 0.46) and 108 girls in Grade 9 (*M*_age_ = 14.43 years, *SD* = 0.40). These participants were drawn from a larger study of developmental influences on support seeking. The younger and older groups represent the typical period when academic stress and digital communication increases (Anniko et al., 2019; Booker et al., 2018). Students with language backgrounds other than English ranged from 31% to 52% of the school population, which is typical of the Sydney area (Australian Bureau of Statistics, 2011). All schools comprised a student body at relative socioeconomic advantage compared with other Australian schools (Australian Curriculum, Assessment and Reporting Authority, 2015).

### Measures

#### Seeking support to cope with academic stressors

Intentions to seek support for academic stressors were assessed with the Adolescent Academic Support-Seeking Scale (AASSS), which was adapted from Skinner et al. (2013). Participants responded to four vignettes depicting academic stressors and rated the intention to seek support from their parents, friends, or digitally on a 5-point scale (1 = not at all and 5 = definitely). A mean score of responses generated a score for each subscale (α_parents_ =.82; α_friends_ =.80; α_digital_ =.84).

#### Depression and anxiety

The youth version of the Depression, Anxiety, and Stress Scale (DASS-Y) (Szabo & Lovibond, 2013) provided an indicator of adolescent mental health on two 8-item subscales assessing symptoms of depression and anxiety. Participants rated how much they had experienced each symptom in the preceding week on a 4-point Likert scale (0 = not true of you and 3 = very true of you), with higher scores indicating increased severity of symptoms (α_depression_ =.90; α_anxiety_ =.87).

### Procedure

This study received institutional ethics approval from the Macquarie University Human Research Ethics Committee (reference number: 5201400949). Principals of schools, students and their parents provided written consent for student participation in the study. Paper-based surveys were completed at the schools, taking an average of 20-minutes to complete.

## Analysis strategy

Analyses were conducted using SPSS 25 and MPlus 8.0 (Muthén & Muthén, 1998–2022). Measurement models were tested for each latent variable, followed by two mediation models. Criteria used to determine a good model fit were comparative fit index (CFI) and Tucker-Lewis index (TLI) greater than .95, root mean square error of approximation (RMSEA) less than .05, and standardised root mean square residual (SRMR) less than .08 (Hu & Bentler, 1999).

### Measurement models

#### Seeking support to cope with academic stressors

One-factor congeneric models were fitted to specify the relationships between individual items and each latent factor in the AASSS. The four-item congeneric model was a good fit for the “digital support-seeking” factor; however, the model did not provide an acceptable fit for the “seeking support from parents” or “seeking support from friends” factors. By deleting one item that was highly similar and retaining a second item with the higher modification index, the three-item models demonstrated good fit and internal reliability for all three factors (Table 1).

**Table 1.** Model fit statistics for measurement models.

Model	RMSEA	SRMR	TLI	CFI	*χ*^*2*^	*df*	*p*	α
Seeking support from parents	.00	.03	1.00	1.00	.82	1	.37	.78
Seeking support from friends	.00	.03	1.00	1.00	.82	1	.37	.77
Digital support-seeking	.00	.01	1.02	1.00	.05	1	.83	.80
Depression	.05	.02	0.98	0.99	7.67^a^	5	.18	.91
Anxiety	.00	.02	1.01	1.00	3.60^a^	5	.61	.84
Full measurement model	.03	.05	.98	.98	170.01^a^	142	.05	-

*Note.* ^a^=Satorra-Bentler Scaled chi-square; χ ^2^ = chi-square; *df* = degrees of freedom; CFI = Comparative Fit and Index; TLI = Tucker-Lewis Index; RMSEA = Root Mean Square Error of Approximation; SRMR = Standardized Root Mean Squared Residual.

#### Depression and anxiety

A one-factor congeneric model, using a Satorra-Bentler χ^2^ post-hoc adjustment to account for the non-normality of the data, did not result in an acceptable fit for the 8-item depression subscale. The modification indices and item wording suggested the presence of two related constructs: one focused on depression and one focused on low enjoyment in-the-moment. Three items measured the second construct and were removed from the model; this created a measure of a single depression construct. The remaining five-item model demonstrated acceptable fit and excellent internal reliability (Table 1).

The one-factor congeneric model for the anxiety subscale, using a Satorra-Bentler χ^2^ post-hoc adjustment, also showed inadequate fit. The *R*^*2*^ values of the three items were low (.32 to .39); with these items removed from the model, the resulting five-item model provided a good fit to the data and internal reliability (Table 1).

#### Full measurement model

A full measurement model was conducted to confirm the factor structure of the latent factors to be included in the structural model. This model provided a good fit to the data (Table 1). Latent factor correlations are shown in Table 2.

**Table 2.** Descriptive statistics and latent factor correlations.

	*M*	*SD*	Latent factor correlations				
Variable			1	2	3	4	5
1. Seeking support from parents	4.08	.86	-				
2. Seeking support from friends	3.35	.95	.186*	-			
3. Digital support-seeking	1.91	.90	−.089	.385**	-		
4. Depression	2.55	3.74	−.168*	−.070	.180*	-	
5. Anxiety	3.35	3.82	−.103	.009	.205**	.646**	-

Note.*=Significant at the 0.05 level; **=Significant at the 0.01 level.

## Results

Descriptive statistics for each latent factor are shown in Table 2. A repeated measures ANOVA with Greenhouse–Geisser correction determined that of the three ways of coping with academic stressors, girls were significantly more likely to report they would seek support from parents than to seek support from friends or use digital support seeking. They were also significantly more likely to seek support from friends rather than use digital support seeking, *F*(1.86, 344.14) = 334.16, *p* < 0.001. On average, girls in this sample reported higher levels of anxiety than depression, *t*(185) = 3.47, *p* <.001

### Structural models

Two structural equation models were tested to examine how digital support-seeking, seeking support from parents, and seeking support from friends were related to depression and anxiety.

#### Model 1

**Figure 1.**
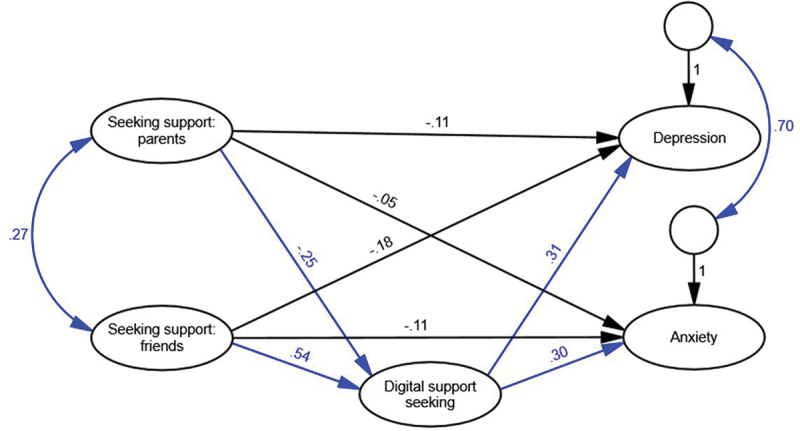
Digital support-seeking as mediator model with standardised path coefficients. Significant paths are shown in blue. Indicators and uniquenesses are omitted for simplicity.

Using digital support-seeking as mediator in Model 1 (Figure 1) provided a good fit to the data. The smaller AIC value of Model 1 indicated that this was the optimal model (Table 3). Direct paths between seeking support from parents or friends and depression or anxiety were negative but not statistically significant. Seeking support from parents negatively predicted digital support-seeking, while seeking support from friends positively predicted digital support-seeking. This suggests that girls who intended to seek support from parents were less likely to use digital support-seeking, but girls who intended to seek support from friends were more likely to use digital support-seeking. Digital support-seeking was in turn a positive predictor of both depression and anxiety, indicating that girls who sought digital support were more likely to report poorer mental health.

**Table 3.** Model fit statistics for the structural models.

	Model 1	Model 2
RMSEA	.03	.04
SRMR	.05	.06
TLI	0.98	0.97
CFI	0.98	0.97
SB-*χ*^*2*^	170.01	181.66
*df*	142	143
*p*	.05	.02
AIC	8425.72	8436.91

*Note.* RMSEA = Root Mean Square Error of Approximation; SRMR = Standardized Root Mean Squared Residual; TLI = Tucker-Lewis Index; CFI = Comparative fit index; SB-*χ^2^ =* Satorra-Bentler Scaled chi-square; *df *= degrees of freedom; AIC = Akaike Information Criteria index.

#### Model 2

**Figure 2.**
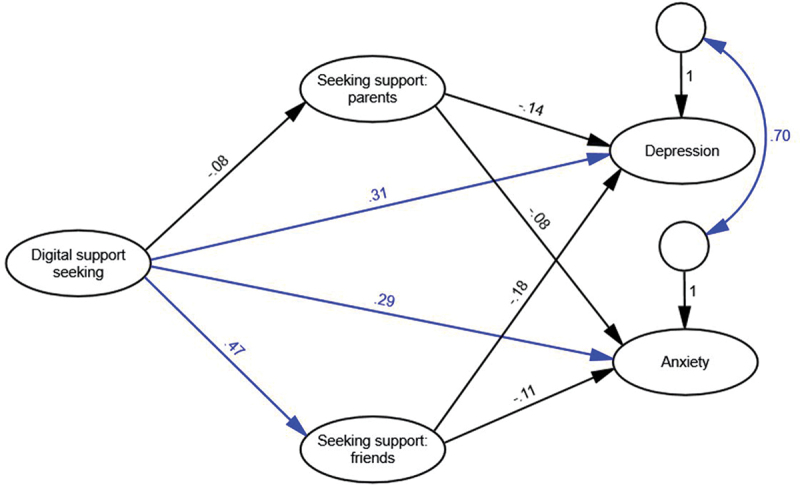
Seeking support from parents and friends as mediator model with standardised path coefficients. Significant paths are shown in blue. Indicators and uniquenesses are omitted for simplicity.

A competing model (Figure 2), in which seeking support from parents and friends mediated the relationship between digital support-seeking and mental health also provided a good fit for the data (Table 3). However, inspection of the significant paths revealed that seeking support from parents was not significantly related to either digital support-seeking or either mental health variable. Seeking support from friends did mediate the relationship between digital support-seeking and mental health, such that direct positive relationships between digital support-seeking and poorer mental health were not present if girls also sought support from their friends.

## Discussion

This study explored relationships between adolescent girls’ digital and traditional support-seeking intentions for academic stressors and their experiences of depression and anxiety. We found positive but non-significant relations between seeking support from parents and friends and better mental health but positive and significant relations between digital support-seeking and poorer mental health. Further, digital support-seeking mediated the relationship between support-seeking from parents and friends and both depression and anxiety, such that benefits of support-seeking from parents and friends were reduced if adolescent girls also sought digital support.

A major finding of this study was that intentions to seek online support to cope with academic stressors were related to higher levels of depression and anxiety. This adds to a growing body of evidence that suggests informal digital support-seeking is related to poorer mental health in adolescents (Duvenage et al., 2020; Frison & Eggermont, 2015). Thus, although support-seeking is viewed as an adaptive way of coping with everyday stressors (Arbel et al., 2018), doing so online may be a problematic way of coping with academic stress. As adolescents may seek emotional support due to experiencing difficulties with schoolwork, and digital emotional support-seeking is associated with increased worry and uncertainty (Rains et al., 2016), this could contribute to greater depression and anxiety. For academic stressors in particular, the constant contact available via digital communication may allow adolescents to turn to peers and for answers to questions rather than solving problems independently (Mackenzie et al., 2020). These behaviours are unlikely to improve the help-seeker’s understanding of schoolwork, contributing additional stress to explain poorer mental health.

A novel contribution of this study was the exploration of digital support-seeking intentions alongside intentions to seek support from parents and friends. Intentions to seek support from parents were negatively related to digital support-seeking, implying that girls with parental support do not go online to access support. This aligns with previous research showing a negative relationship between parent relationship quality and adolescent digital communication (Foerster & Röösli, 2017). As such, seeking support from parents may be protective against non-adaptive forms of digital support-seeking for academic stressors, while providing an adaptive way of coping with stress. Previous research confirms the positive implications of parent support for adolescent wellbeing (Heerde & Hemphill, 2018).

A second novel contribution was the finding that digital support-seeking mediated the relationship between support-seeking from friends and depression and anxiety. While the direct effects between intentions to seek support from friends and depression and anxiety were non-significant, the indirect effects through digital support-seeking were significant and positive. This suggests that girls who turn to friends for academic support are also more likely to seek digital support; in turn, this is related to higher levels of depression and anxiety. The practical implication of this finding is that parents and teachers should be wary of encouraging adolescent girls to discuss academic concerns online; this may be associated with non-adaptive support seeking behaviours and poor mental health outcomes.

Over and above the limitations associated with a cross-sectional study design, there are other limitations specific to this study. We chose to focus specifically on female participants, meaning that our findings should not be generalised to adolescent boys. Given the emerging evidence for differences in relationships between online communication and wellbeing for males and females (e.g., Brandtzæg, 2012; Frison & Eggermont, 2016), we recommend that future research considers specific studies with male and non-binary participants to examine the implications of digital support seeking for males and non-binary adolescents. Another limitation to note is that our coping vignettes measured intentions to cope rather than actual coping responses. While vignettes are an established way of measuring coping that allows for the standardisation of stressors across participants (Pitzer & Skinner, 2017; Skinner et al., 2013), it is possible that some students’ actual coping responses may differ from their intended responses (Zimmer-Gembeck, 2015). Given our findings that a range of coping intentions predict students’ mental health outcomes, we suggest that both coping intentions and coping behaviours are important. Finally, we did not assess who adolescents intended to seek support from online. In other research, these same adolescents reported that digital interactions were with in-person friends (Mackenzie et al., 2020); however, it is possible that adolescents seek online support from other acquaintances too. Given our finding that digital support seeking is related to increased depression and anxiety, we recommend that future research test this finding in different contexts by examining the different sources of support that adolescent girls seek support from online and the quality of this support.

Notwithstanding these limitations, this study contributes to our understanding of how adolescent girls' intended support-seeking behaviours are concurrently related to depression and anxiety symptoms. The findings suggest that digital support-seeking is a problematic way of coping with academic stress, demonstrating relationships with higher indicators of depression and anxiety. Further, seeking support from parents can tentatively be considered a protective factor due to its negative relationship with digital support-seeking. Given the salience of academic stressors for adolescents, the findings suggest that parents and teachers should be wary of maladaptive outcomes when adolescents seek help online for academic concerns. Future research should consider if and how digital support-seeking can be employed as a useful coping mechanism for academic stressors.

## Data Availability

Due to the nature of this research, participants of this study did not agree for their data to be shared publicly.
